# Aberrant Expression and Secretion of Heat Shock Protein 90 in Patients with Bullous Pemphigoid

**DOI:** 10.1371/journal.pone.0070496

**Published:** 2013-07-30

**Authors:** Stefan Tukaj, Konrad Kleszczyński, Katerina Vafia, Stephanie Groth, Damian Meyersburg, Piotr Trzonkowski, Ralf J. Ludwig, Detlef Zillikens, Enno Schmidt, Tobias W. Fischer, Michael Kasperkiewicz

**Affiliations:** 1 Department of Dermatology, University of Lübeck, Lübeck, Germany; 2 Department of Clinical Immunology and Transplantology, Medical University of Gdańsk, Gdańsk, Poland; CWRU/UH Digestive Health Institute, United States of America

## Abstract

The cell stress chaperone heat shock protein 90 (Hsp90) has been implicated in inflammatory responses and its inhibition has proven successful in different mouse models of autoimmune diseases, including epidermolysis bullosa acquisita. Here, we investigated expression levels and secretory responses of Hsp90 in patients with bullous pemphigoid (BP), the most common subepidermal autoimmune blistering skin disease. In comparison to healthy controls, the following observations were made: (i) Hsp90 was highly expressed in the skin of BP patients, whereas its serum levels were decreased and inversely associated with IgG autoantibody levels against the NC16A immunodominant region of the BP180 autoantigen, (ii) in contrast, neither aberrant levels of circulating Hsp90 nor any correlation of this protein with serum autoantibodies was found in a control cohort of autoimmune bullous disease patients with pemphigus vulgaris, (iii) Hsp90 was highly expressed in and restrictedly released from peripheral blood mononuclear cells of BP patients, and (iv) Hsp90 was potently induced in and restrictedly secreted from human keratinocyte (HaCaT) cells by BP serum and isolated anti-BP180 NC16A IgG autoantibodies, respectively. Our results reveal an upregulated Hsp90 expression at the site of inflammation and an autoantibody-mediated dysregulation of the intracellular and extracellular distribution of this chaperone in BP patients. These findings suggest that Hsp90 may play a pathophysiological role and represent a novel potential treatment target in BP.

## Introduction

Bullous pemphigoid (BP) is the most common autoimmune subepidermal blistering skin disease, usually occurring in the elderly, characterized by autoantibodies to the hemidesmosomal components BP180 and BP230 [1]. The pathogenic relevance of autoantibodies against BP180, which lead to a cascade of inflammatory events resulting in subsequent blister formation at the dermal-epidermal junction, has been conclusively shown ex vivo and in experimental animal models. Detection of circulating IgG autoantibodies against the immunodominant region of BP180 (BP180 NC16A) facilitates diagnosis of BP and monitoring of these autoantibodies is helpful to evaluate the efficacy of treatment as they correlate with disease activity [1], [2].

Heat-shock proteins (Hsps) are a family of ubiquitous molecular chaperones essential for protein folding and transport within the cell, with Hsp90 being one of the most abundant proteins in the eukaryotic cell. It participates in structural maturation and conformational regulation of a number of signaling molecules and transcription factors and constitutes up to 1–2% of the cellular protein under physiological conditions. Its expression is several-fold enhanced in response to stresses placed upon the cell, e.g. in the context of ultraviolet radiation, heavy metals, malignancies, and inflammation. While Hsp90 resides primarily in the cytoplasm, it can also be released to extracellular compartments in response to such stressful conditions or upon cell death [3].

Hsp90 has also been increasingly recognized to play important roles in antigen presentation, activation of lymphocytes and macrophages, and activation and maturation of dendritic cells, suggesting that Hsp90 may be involved in the pathophysiology of inflammatory diseases such as autoimmune diseases [4]. Recent data from animal models of experimentally induced autoimmune encephalomyelitis [5], rheumatoid arthritis [6], [7] and systemic lupus erythematosus [8], [9] indicate that pharmacological inhibition of Hsp90 may represent a novel potential treatment of autoimmune disorders. Very recently, using mice with experimental epidermolysis bullosa acquisita, we were able to show that inhibition of Hsp90 is associated with amelioration of clinical signs, suppression of autoantibody production and reduction of inflammatory skin infiltrate in this autoimmune bullous skin disease [10].

This work aimed at investigating expression levels and secretory responses of Hsp90 in skin and blood of patients with BP in comparison to healthy subjects and a control cohort of autoimmune bullous disease patients with pemphigus vulgaris, studies that may shed light on whether Hsp90 could play a role in the pathogenesis of human BP and represent a potential novel target for treatment of these patients.

## Materials and Methods

### Declarations

The investigations were conducted under approval from the Ethics Committee of the University of Lübeck and the Ethics Committee of the University of Gdańsk and with written informed consent.

### Patients

The diagnosis of BP in our study patients, who were admitted in the Department of Dermatology of the University of Lübeck between 2006 and 2012, was based on typical clinical findings as well as on detection of linear deposits of IgG and/or C3 at the dermal-epidermal junction and circulating IgG autoantibodies against BP180 NC16A by direct immunofluorescence microscopy and enzyme-linked immunosorbent assay (ELISA; Euroimmun, Lübeck, Germany), respectively. Overall, 23 BP patients (mean age 73.3±16.2 years, 13 females, 10 males) and 40 age- and gender-matched healthy volunteers (mean age 69.2±17.4 years, 24 females, 16 males) were included to this study. An analysis of sera from 12 BP patients with initially high levels of circulating anti-BP180 NC16A autoantibodies (mean 1309±2821 U/ml, range 70 to 5543 U/ml) and mainly active disease (7 patients with active blistering and 5 in complete remission on therapy) as well as from 29 healthy volunteers was performed to detect levels of circulating Hsp90. Sera from 10 patients with pemphigus vulgaris (mean age 56.1±11.9 years, 5 females, 5 males) with initially high levels of circulating anti-desmoglein 3 (mean 1159.1±1835.2 U/ml, range 0 to 6201 U/ml) and 1 autoantibodies (mean 148.4±312.9 U/ml, range 0 to 1000 U/ml) and active disease as well as from 12 corresponding age- and gender-matched healthy volunteers (mean age 56.2±14.45 years, 7 females, 5 males) served as additional controls. Further, another set of sera was analyzed from these BP and pemphigus patients after a mean follow-up period of 29 and 41 weeks, respectively, during which they received immunosuppressive medication leading to a decline in circulating autoantibodies and healing of skin lesions. To detect Hsp90 expression in the skin, perilesional skin biopsies from 6 BP patients with active disease and skin samples from 6 healthy volunteers were analyzed. For cell culture experiments, peripheral blood mononuclear cells (PBMCs) from 5 as yet untreated BP patients with active disease and 5 healthy donors were used.

### Autoantibody Levels

Circulating IgG autoantibodies to BP180 NC16A as well as to desmoglein 1 and 3 were detected by ELISA according to the manufacturer’s instructions (Euroimmun, Lübeck, Germany).

### Hsp90 Detection in Skin

Skin samples were embedded in freezing Cryomatrix formula (Thermo Scientific Ltd., Runcore, Cheshire, UK), snap-frozen in liquid nitrogen, and stored at −80°C. Skin samples were cut into 6-µm-thick sections using Leica CM 3050S research cryostat (Leica Mikrosysteme GmbH, Wetzlar, Germany) and then subjected to immunofluorescence examinations, as described earlier [11]. Detection of Hsp90 was performed by the tyramide signal amplification (TSA) procedure. Briefly, frozen sections of human skin were dried and fixed with ice-cold acetone for 10 min at −20°C and washed 3 times for 5 min each with 1×TNT (pH 7.5). Endogenous peroxides were quenched using 3% H_2_O_2_ for 15 min at room temperature (RT), washed with TNT again, and blocked in incubation sequence: avidin, TNT, biotin, TNT. The pre-incubation was carried out using 2% normal goat serum (NGS; Dako, Inc., Carpinteria, CA, USA) for 30 min at RT. After this, sections were incubated with rabbit polyclonal anti-Hsp90 IgG (1∶400; Abcam, Cambridge, UK) overnight at 4°C in presence of 2% NGS. The slides were washed 3 times with TNT and treated in presence of 2% NGS with secondary biotin-labeled goat anti-rabbit IgG (1∶400; Jackson ImmunoResearch Laboratories, Inc., West Grove, PA, USA) for 45 min at RT. Sections were washed again with TNT buffer and staining was developed using TSA Fluorescein System (Perkin Elmer, Boston, MA, USA). First, slides were incubated with horseradish-peroxidase-conjugated streptavidin (1∶100) for 30 min at RT, washed 3 times with TNT, and labeled using fluorescein tyramide reagent (1∶50) for 5 min at RT. Samples were counterstained with DAPI solution (1 µl ml^−1^) for 1 min, washed with TNT, and mounted in Fluoromount-G mounting medium (SouthernBiotech, Birmingham, AL, USA). All fluorescent images were obtained using the Keyence BZ-8000 inverted fluorescence microscope (Keyence GmbH, Neu-Isenburg, Germany). The fluorescence intensity of the epidermal layer of stained skin sections was determined by densitometry measurements using image J 1.38d software (National Institute of Health, USA).

### Hsp90 Detection in serum and Cell Culture

Hsp90 was evaluated in serum as well as in conditioned medium or PBMC and human keratinocyte (HaCaT) cell extracts by a commercial Hsp90α ELISA (Enzo Life Science, Lörrach, Germany), following the manufacturer’s instructions. The amount of the protein was calculated according to standard curve prepared from the protein included in the kit.

### TGF-α Detection

TGF-α in serum was measured by a commercial TGF-α ELISA (Abcam, Cambridge UK), following the manufacturer’s instructions.

### PBMC Cell Culture

PBMCs were isolated from venous blood by Ficoll-Paque (GE Healthcare, Munich, Germany) gradient centrifugation and cultured as described previously with minor modification [12]. Briefly, PBMCs were washed twice in PBS and freshly isolated PBMC were subjected to lysis buffer or re-suspended to 1×10^6^ ml^−1^ of medium (RPMI 1640 supplemented with 10% fetal calf serum, 2 mM L-glutamine, and penicillin/streptomycin). The cells were treated by LPS (1 µg ml^−1^; Sigma Aldrich, Munich, Germany) and CpG (3 µg ml^−1^; Hycult Biotechnology, Uden, the Netherlands) or TNF-α (20 ng ml^−1^; R&D Systems Inc., Minneapolis, USA) in 24-well plates. After 18 hours, culture medium was collected and stored in −20°C for further analysis.

### Immunoblotting

PBMCs lysates (20 µg per line) were separated by 10% SDS-PAGE (Mini Protean II, BioRad) and transferred to nitrocellulose membrane. The membrane was blocked with 3% milk in PBS for 2 hours, followed by incubation with rabbit polyclonal antibodies against protein phosphatase 5 (PP5; 1∶100; Abcam), protein kinase A (PKA) αβ (1∶1000; Abcam), and β-Actin (1∶1000; Sigma) at RT for 2 hours. Horseradish peroxidase-conjugated gout anti-rabbit antibodies (1∶1000; Dako, Hamburg, Germany) were used as secondary antibodies. The bands were visualized using Amersham ECL Plus Western Blotting Detection Reagents (GE Healthcare, Munich, Germany). PP5 and PKA concentrations were expressed relative to the β-Actin level using densitometry measurements.

### Purification of Total IgG and Anti-BP180 NC16A IgG Autoantibodies

Total IgG from a BP patient or healthy blood donor was isolated using Protein G Sepharose Fast Flow affinity column chromatography (GE Healthcare, Munich, Germany) as described previously [13]. Antibodies were eluted with 0.1 M glycin buffer (pH 2.8), neutralized with Tris-HCL (pH 9.0), and concentrated under extensive washing with PBS using Amicon Ultra-15 filters (Millipore, Bradford, MA, USA). Antibodies specific to NC16A were affinity-purified from IgG of BP patients using tetrameric NC16A coupled on sepharose. Specific antibodies were eluted as described above. The IgG of the flow-through (NC16A-depleted) fraction was also used for the experiments. Reactivity of pathogenic IgG was analyzed by indirect immunofluorescence microscopy on HaCaT cells and by Anti-BP180-NC16A-4X ELISA diagnostic kit (Euroimmun), respectively.

### HaCaT Cell Culture

HaCaT cells were cultured in serum-free keratinocyte growth medium (Promo Cell, Heidelberg, Germany) at 37°C in 5% CO_2_ atmosphere. Cells were seeded on 12-well plates (Greiner Bio-One, Solingen, Germany) and grown to 80% confluence. Cells were incubated with total IgG of normal human serum from a healthy blood donor, total IgG from a BP patient, affinity-purified anti-BP180 NC16A IgG, NC16A-depleted anti-BP180 IgG, intact sterile sera of BP patients, or sera from healthy blood donors. After 24 hours of incubation, the supernatants were taken and the cells were washed twice with PBS, harvested, washed with PBS again, lysed, and stored for further analysis at −20°C.

### Statistical Analysis

Data were analyzed using two-tailed Student’s t-test, one-way analysis of variance (ANOVA) or Spearman’s rank correlation test using Sigma plot 12.0 (Systat Software, Chicago, IL). A *P*-value <0.05 was considered statistically significant.

## Results

### Hsp90 is Highly Expressed in the Skin of BP Patients

**Figure 1:**
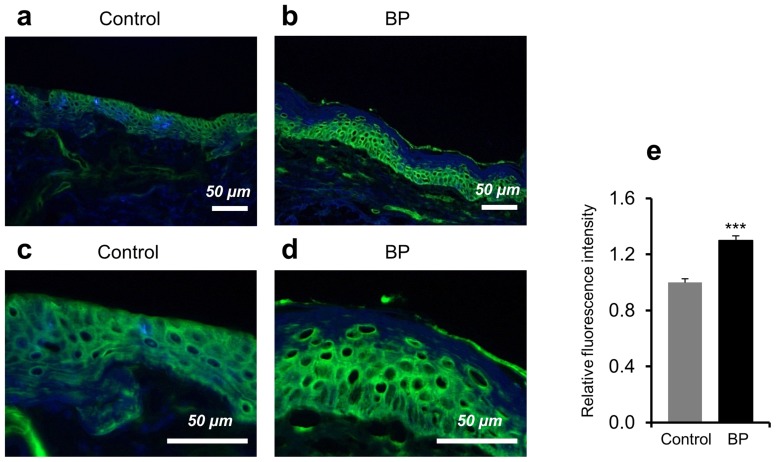
High expression of Hsp90 in the epidermis of bullous pemphigoid patients. Hsp90 expression was analyzed in perilesional skin biopsies of bullous pemphigoid (BP) patients and in skin sections of healthy controls by fluorescent immunohistochemistry. (**a, b**) Representative immunofluorescent and DAPI-counterstained (in blue) skin samples showed a stronger intracellular Hsp90 expression (in green) mainly localized to the epidermal layer of the skin of a BP patient compared to control skin. Magnification: x200. (**c, d**) Higher magnification (x600) of the respective skin samples. (**e**) Relative mean epidermal Hsp90 fluorescence intensity by densitometry measurements. Values are mean ± SEM of 6 BP patients and 6 controls. ***P<0.001.

Using fluorescent immunohistochemistry and densitometry measurements, we found that expression of Hsp90 was significantly increased in the epidermis of perilesional skin biopsies from BP patients (*n* = 6) as compared with skin samples of healthy controls (*n* = 6, *P*<0.001, Figure 1a–e). In contrast, Hsp90 was only weakly detected in the dermal layer with no distinguishable cellular localization in all skin samples (Figure 1a–d).

### Serum Levels of Hsp90 are Decreased in BP Patients and Inversely Associated with Anti-BP180 NC16A Autoantibody Levels

**Figure 2:**
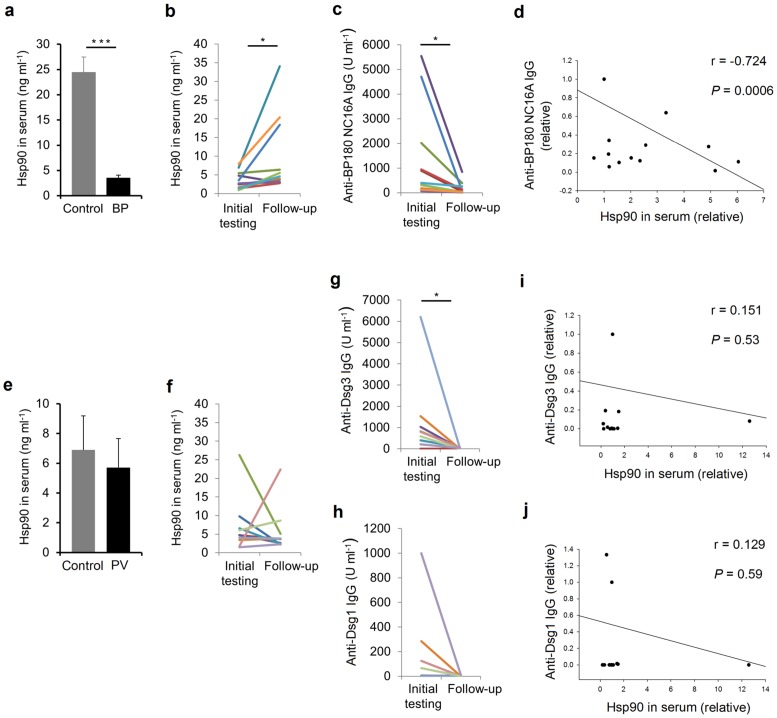
Decreased Hsp90 serum levels and their inverse association with anti-BP180 NC16A autoantibodies in bullous pemphigoid patients. Circulating Hsp90 was evaluated in serum of bullous pemphigoid (BP) patients and controls (healthy subjects and pemphigus vulgaris [PV] patients) by enzyme-linked immunosorbent assay. (**a**) Mean serum Hsp90 levels were comparatively lower in BP patients than in healthy controls. (**b**) Intraindividual serum levels of Hsp90 in BP patients showed an increase after a mean follow-up time of 29 weeks of immunosuppressive treatment. (**c**) Rising levels of circulating Hsp90 were paralleled by a reduction in individual serum anti-BP180 NC16A IgG autoantibodies. (**d**) A significant inverse correlation was observed between Hsp90 serum levels and circulating BP autoantibodies. (**e**) Mean serum Hsp90 levels were comparable between PV patients and corresponding healthy controls. (**f**) Intraindividual serum levels of Hsp90 in PV patients showed no consistent trend towards an increase or decrease, while serum (**g**) anti-desmoglein 3 (Dsg3) and (**h**) 1 (Dsg1) IgG autoantibodies declined after a mean follow-up time of 41 weeks of immunosuppressive treatment. (**i, j**) No significant correlation between Hsp90 serum levels and circulating PV autoantibodies could be recorded. Values are mean ± SEM of 12 BP patients, 10 PV patients and 12–29 healthy controls. *P<0.05, ***P<0.001.

We observed that sera of BP patients (*n* = 12) contained approximately 7-fold lower levels of circulating Hsp90 in comparison with serum samples from healthy individuals (*n* = 29) as measured by ELISA (*P*<0.001; Figure 2a). Interestingly, intraindividual serum levels of Hsp90 increased 2.6-fold (*P*<0.05, Figure 2b) in parallel with a decline in circulating anti-BP180 NC16A autoantibodies (from mean 1309±2821 U/ml to mean 163±1677 U/ml; *P*<0.05, Figure 2c) as well as healing of skin lesions (of the 7 patients with active blistering at initial testing, 5 went into complete and 2 into partial remission, while the remaining 5 patients in complete remission on therapy at initial testing remained free of skin lesions) after a mean follow-up time of 29 weeks of immunosuppressive treatment, although still being lower compared to healthy individuals (data not shown). This inverse relationship between Hsp90 serum levels and circulating autoantibodies was found to be statistically significant (*r* = −0.724, *P* = 0.0006, Figure 2d). A significant inverse correlation was also calculated for Hsp90 serum levels and the clinical status, but only for the subgroup of BP patients presenting with active disease at initial testing (*r* = −0.613, *P* = 0.02). In contrast, sera from control patients with pemphigus vulgaris (*n* = 10) showed similar levels of circulating Hsp90 to those of serum samples from corresponding healthy persons (*n* = 12, *P* = 0.16, Figure 2e). In addition, no consistent trend towards an increase or reduction of circulating Hsp90 was found in these pemphigus patients after a mean follow-up time of 41 weeks of immunosuppressive treatment (*P* = 0.36, Figure 2f), during which a decrease in circulating anti-desmoglein 3 (from 1159.1±1835.2 U/ml to mean 27.7±28.4 U/ml; *P*<0.05, Figure 2g) and 1 autoantibodies (from mean 148.4±312.9 U/ml to mean 1.4±2.9 U/ml; *P* = 0.08, Figure 2h) as well as healing of skin lesions (all 10 patients with active blistering went into complete remission) was observed. This resulted in no correlations between Hsp90 serum levels and circulating anti-desmoglein 3 (*r* = 0.151, *P* = 0.53, Figure 2i) and 1 autoantibodies (*r* = 0.129, *P* = 0.59, Figure 2j) or the clinical status of these control patients (*r* = 0.185, *P* = 0.434).

### Hsp90 is Highly Expressed in and Restrictedly Released from PBMCs of BP Patients

**Figure 3:**
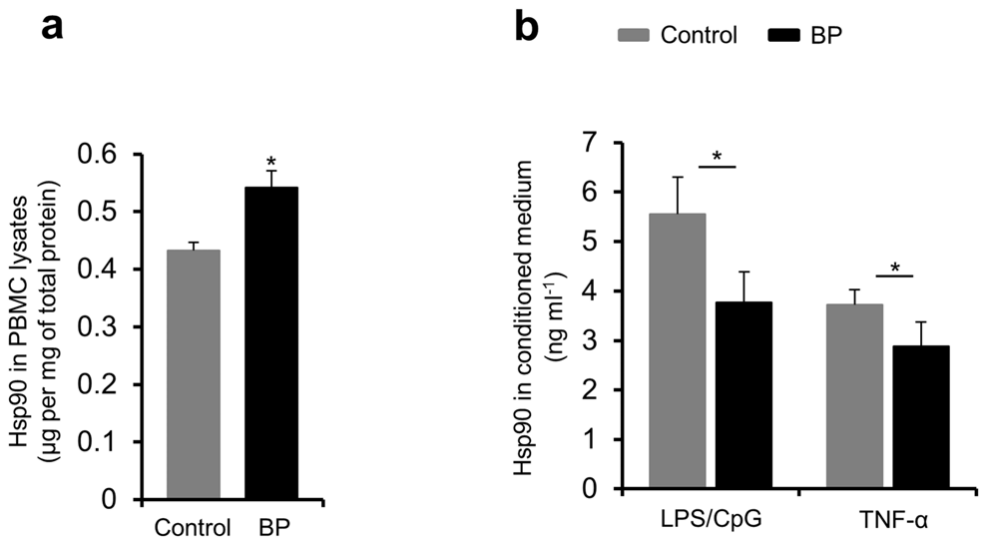
High expression of Hsp90 and its compromised secretion by peripheral blood mononuclear cells of bullous pemphigoid patients. Hsp90 was detected in whole cell lysates of freshly isolated peripheral blood mononuclear cells (PBMCs) as well as in conditioned medium of PBMC cultures of bullous pemphigoid (BP) patients and healthy controls by enzyme-linked immunosorbent assay. (**a**) The mean intracellular Hsp90 content of PBMCs was comparatively higher in BP patients. (**b**) The mean extracellular Hsp90 content of PBMC cultures was comparatively lower in BP patients after 18-hour stimulation with LPS/CpG and TNF-α. Values are mean ± SEM of 5 BP patients and 5 controls. *P<0.05.

Freshly isolated PBMCs of BP patients (*n* = 5) showed a significantly increased intracellular Hsp90 content compared to PBMCs from healthy volunteers (*n* = 5) as measured by ELISA with cell lysates (*P*<0.05, Figure 3a). To investigate if both the higher intracellular Hsp90 content in BP PBMCs and the lower serum levels of Hsp90 in BP patients may be a result of compromised secretion of this protein into the extracellular space, PBMCs of BP patients (*n* = 5) and healthy controls (*n* = 5) were cultured and stimulated by the proinflammatory agents LPS/CpG and TNF-α for 18 hours, followed by Hsp90 detection in conditioned medium using ELISA. In fact, secretion of Hsp90 by PBMCs of BP patients was significantly lower compared to PBMCs from healthy controls (*P*<0.05, Figure 3b).

### Hsp90 is Potently Induced in and Restrictedly Secreted from Keratinocytes by BP Serum and Isolated Anti-BP180 NC16A IgG, Respectively

**Figure 4:**
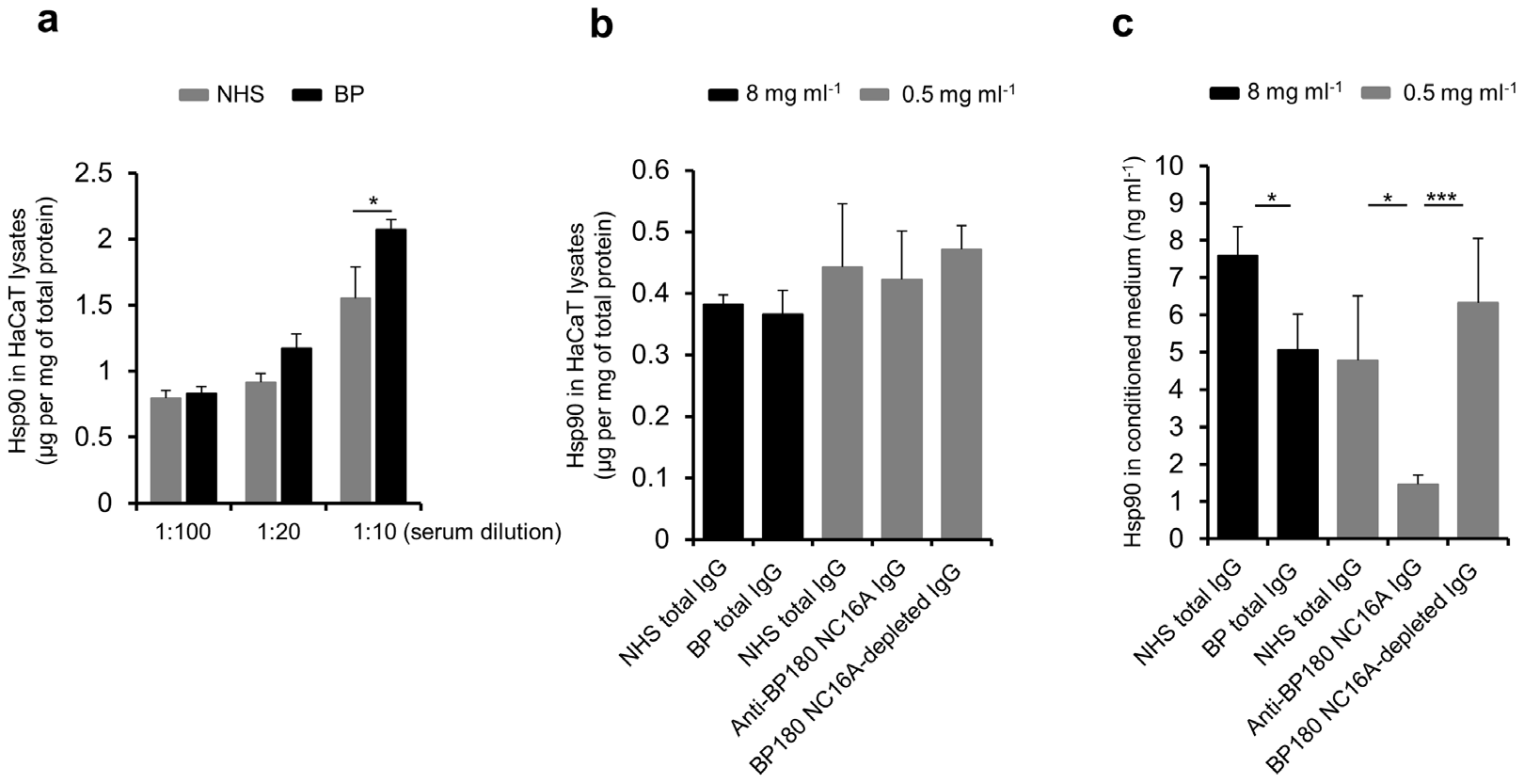
Potent induction of Hsp90 expression in and its compromised release from keratinocytes by BP serum and isolated anti-BP180 NC16A IgG, respectively. (**a**) Human keratinocyte (HaCaT) cells were incubated with normal human serum (NHS) of healthy donors and serum of bullous pemphigoid (BP) patients over 24 hours. With increasing serum concentration a comparatively stronger intracellular upregulation of Hsp90 was induced by BP serum as measured by enzyme-linked immunosorbent assay (ELISA) with HaCaT cell lysates. Values are mean ± SEM of 2 independent experiments of 3 BP patients and 3 controls. *P<0.05. (**b**) This finding was not dependent on the presence of BP autoantibodies, as a 24-hour stimulation of HaCaT cells with total BP IgG or affinity-purified anti-BP180 NC16A IgG from a BP patient failed to reproduce such effect when compared with NHS IgG from a healthy donor as measured by ELISA with HaCaT cell lysates. Values are mean ± SEM of 4 independent experiments. (**c**) Total BP IgG or affinity-purified anti-BP180 NC16A IgG from a BP patient, but not BP180 NC16A-depleted IgG, significantly inhibited the secretion of Hsp90 compared to NHS IgG from a healthy donor as measured by ELISA with conditioned medium of 24-hour stimulated HaCaT cells. Values are mean ± SEM of 4 independent experiments. *P<0.05, ***P<0.001.

HaCaT cells were incubated with serum samples of healthy donors (*n* = 3) and BP patients (*n* = 3) over 24 hours. Both normal and BP patients’ sera were able to induce the expression of Hsp90 in these cells. However, with increasing serum concentration a significantly stronger intracellular upregulation of this protein was observed using BP serum as measured by ELISA with HaCaT cell lysates (*P*<0.05, Figure 4a), supporting our above mentioned skin immunohistochemistry results of a more intense Hsp90 expression within the epidermis of BP patients compared to controls.

Interestingly, this effect could not be reproduced by a 24-hour stimulation of HaCaT cells with total BP IgG or affinity-purified anti-BP180 NC16A IgG compared to normal control IgG (Figure 4b), suggesting that serum inflammatory components other than BP autoantibodies lead to accumulation of Hsp90 within keratinocytes in these patients.

Although there was no difference between BP autoantibodies and normal human serum IgG with regard to induction of Hsp90 in HaCaT cells, total BP IgG or affinity-purified anti-BP180 NC16A IgG from a BP patient significantly inhibited the secretion of Hsp90 compared to control IgG from a healthy donor as measured by ELISA with conditioned medium of 24-hour stimulated HaCaT cells (*P*<0.05, Figure 4c). This impaired cellular release of Hsp90 was found to be restricted to the presence of pathogenic autoantibodies against the immunodominant region of BP180, since anti-BP180 NC16A-depleted IgG was no longer capable of inhibiting the secretion of Hsp90 from HaCaT cells (Figure 4c).

### Impaired Levels of Secreted Hsp90 are Independent of TGF-α, PP5 and PKA

**Figure 5:**
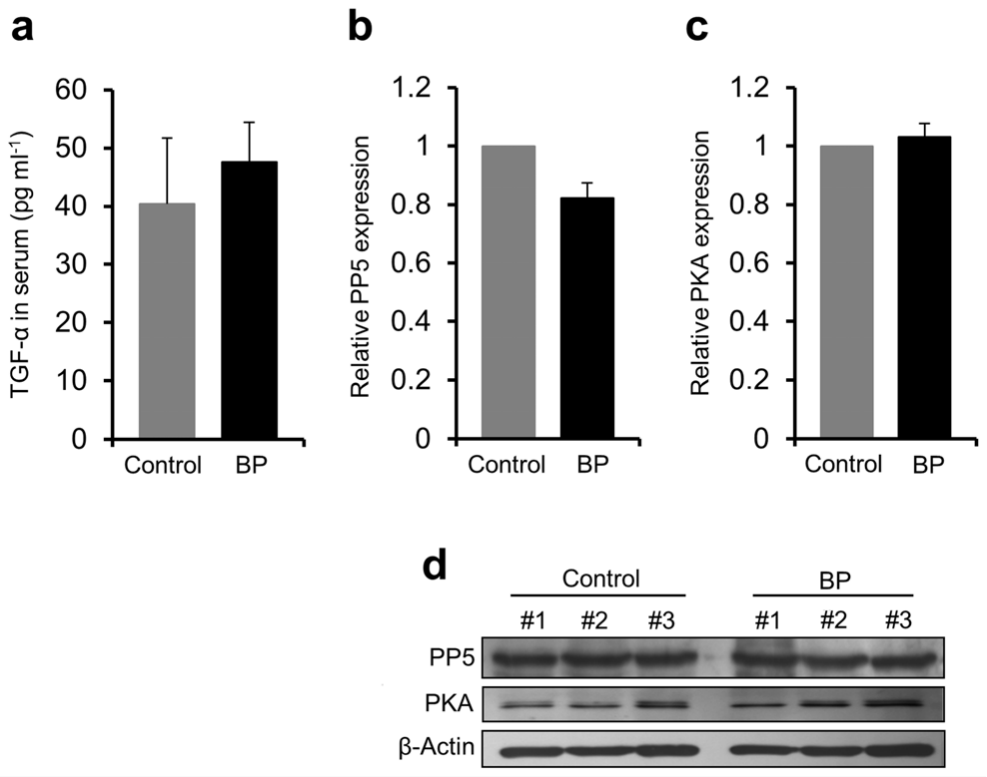
No contribution of TGF-α, protein phosphatase 5 or protein kinase A to decreased levels of secreted Hsp90. Expression of (**a**) TGF-α, (**b**) protein phosphatase 5 (PP5) and (**c**) protein kinase A (PKA) was analyzed in serum (TGF-α) and peripheral blood mononuclear cells (PBMC) lysates (PP5 and PKA) of bullous pemphigoid (BP) patients and healthy controls by enzyme-linked immunosorbent assay and western blotting, respectively, and revealed no significant difference between both study groups. (**d**) Mean PP5 and PKA concentrations were expressed relative to the β-Actin level using densitometry measurements of immunoblots. Values are mean ± SEM of 3–9 BP patients and 3–6 controls.

TGF-α, PP5 and PKA were previously described to be involved in the regulatory mechanism of Hsp90 secretion [14], [15]. However, ELISA measurements of TGF-α serum levels as well as western blot analyses of expression levels of PP5 and PKA in PBMCs of our BP patients (*n* = 3–9) and healthy controls (*n* = 3–6) revealed that these factors showed no significant difference between both study groups (Figure 5a–d).

## Discussion

The present study suggests that Hsp90 is involved in the pathology of BP as both altered expression levels and restricted secretory responses of Hsp90 were found in these patients.

Cells of both epidermis and dermis constitutively express different Hsps, including Hsp90 [16], and some of them have been described to be upregulated in a variety of inflammatory skin conditions including UV-, hyperthermia- and heavy metal-provoked skin reactions [17]–[19], psoriasis [20], atopic dermatitis [21], systemic lupus erythematosus [22], lichen planus [23], Behçet’s disease [24], and graft-versus-host disease [25]. Except for mucous membrane pemphigoid, in which Hsp90 was found to be highly upregulated in the conjunctiva of affected patients [26], data on expression levels of this chaperone in autoimmune bullous skin diseases are lacking. We therefore first aimed at investigating, whether Hsp90 is also overexpressed in the skin of BP patients. We found a significantly stronger intracellular Hsp90 expression in the skin of BP patients compared to normal skin control specimens. This high expression was rather confined to the epidermal layer confirming previous investigations demonstrating that Hsp90 is expressed predominantly within the epidermis [16], [18]. We were able to reproduce this finding of an intracellular Hsp90 increase using HaCaT cells incubated with BP serum. Interestingly, we found that this result was not dependent on the presence of BP autoantibodies, suggesting that other serum inflammatory components like for example cytokines may lead to accumulation of Hsp90 within keratinocytes in these patients. The induction of Hsp90 within skin cells may be a result of a protective response in an attempt to refold damaged polypeptides, but also suggests the potential for interaction with other mechanisms involved in the pathogenesis of the disease. In this regard, it is worth noting that Hsp90 has been revealed to be critical for intracellular signaling that participates in inflammatory cytokine production, including IL-6 and IL-8 [3], [4], [27], [28]. Several lines of evidence underscore the importance of both of these proinflammatory cytokines as well as NF-kappa B, their major transcriptional factor, in the pathogenesis of BP. Release of IL-6 and IL-8 from keratinocytes is believed to represent one of the earliest pathophysiological steps in BP, leading to chemotaxis-induced granulocyte recruitment essential for blister formation [1], [2], [13], [29]. As specific pharmacological inhibition of Hsp90 has previously been described to reduce stability and translation of the transcripts of both of these cytokines partly due to deactivation of NF-kappa B [27], [28], pharmacological blockade of Hsp90 could therefore represent a promising therapeutic approach interfering with this key inflammatory cascade event in BP.

We observed an increase in the intracellular content of Hsp90 not only in keratinocytes, but also in PBMCs of BP patients with active disease when compared with PBMCs from healthy controls. Similarly, an overexpression of Hsp90 in PBMCs was observed in different rheumatic diseases including systemic lupus erythemtosus, dermato/polymyositis, primary Sjogren’s syndrome, systemic sclerosis, Behcet’s syndrome, and multiple sclerosis [30]. In systemic lupus erythematosus, the elevated levels of Hsp90 in PBMCs were correlated with increased levels of circulating IL-6 [31]. Since serum levels of IL-6 have been described to correlate with disease activity in both systemic lupus erythematosus and BP [32], [33], further studies of a more detailed analysis of the possibly pathophysiologically relevant IL-6-Hsp90 interplay in BP patients are warranted.

Traditionally, Hsps are regarded as intracellular molecules. However, upon cell death or in response to a number of stressful conditions, Hsps can be released into extracellular compartments [3], [4]. An increased Hsp90 release from PBMCs has previously been reported in patients with active systemic lupus erythematosus and a significantly higher amount of soluble Hsp90 was observed in sera of patients with the chronic inflammatory disorder atherosclerosis compared with healthy subjects’ sera [34], [35]. In contrast, we found that serum levels of Hsp90 were significantly lower in our patients compared to healthy controls. In addition, the Hsp90 supernatant content of stimulated PBMCs and HaCaT cells was lower compared to controls, indicating that the release of Hsp90 from blood- as well as skin-derived cells is compromised and therefore presumably responsible for the observed lower serum levels of Hsp90 in BP patients.

Although the exact mechanisms of Hsp90 release form cells are generally unknown, inhibition of Hsp90 secretion from BP cells seemed not to depend on altered TGF-α, PP5 and PKA expressions, all of which have previously been described to be involved in the Hsp90 secretory process [14], [15], but on the presence of specific BP autoantibodies. We found that HaCaT cells showed an impaired liberation of Hsp90 after incubation with total BP IgG or affinity-purified anti-BP180 NC16A IgG, but no longer with BP180 NC16A-depleted IgG. This may at least in part explain the observed finding of a resurgence of Hsp90 serum levels during the course of the disease in our study patients when anti-BP180 NC16A autoantibodies levels decreased in parallel with healing of skin lesions.

In contrast to BP, neither aberrant levels of circulating Hsp90 nor any correlation of this protein with serum anti-desmoglein autoantibodies or clinical status was found in a control cohort of patients with pemphigus vulgaris, a different autoimmune blistering disease characterized by an autoimmune response against desmosomal structures of the skin. Together with our above mentioned experimental findings, this observation further suggests a rather specific role of Hsp90 in anti-BP180 autoantibody-driven disease pathology and indicates a differential contribution of this protein to inflammatory processes of autoimmune skin disorders.

The questions why the extracellular Hsp90 content of BP patients is lower compared to healthy controls and why BP autoantibodies negatively regulate Hsp90 release remain largely unknown. However, there could be 3 mutually nonexclusive explanations for this observation.

First, a mechanistic effect compensating the high intracellular load of Hsp90 may be possible. On the other hand, although extracellular Hsp90 is generally believed to be proinflammatory by inducing cytokines and enhancing the antigenicity of autoantigens through modulations of antigen presentation [4], secreted endoplasmatic reticulum-derived Hsp90 has also been described to stimulate anti-inflammatory regulatory T cell responses and to regulate the generation of tolerogenic plasmacytoid dendritic cells [36]–[38], thereby inducing a negative feedback control of inflammation. Abrogation of this latter effect by lowered amounts of extracellular Hsp90 may therefore also be reasonable and account for the disease state of the BP patients. We thirdly speculate that the lack of extracellular Hsp90, which has also been recently recognized as major factor of wound healing [39], could contribute to an impaired healing of blister-associated erosions and therefore be responsible for the maintenance of skin lesions in BP patients.

**Figure 6:**
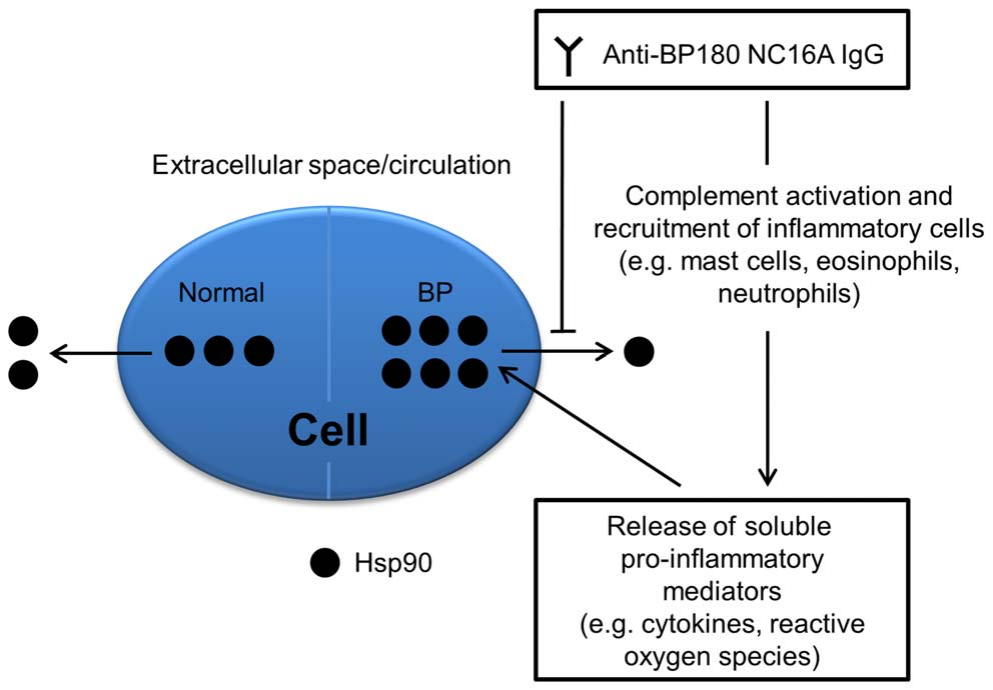
Proposed model for aberrant expression of Hsp90 in and its restricted release from keratinocytes in bullous pemphigoid. Under physiological conditions, ubiquitously and constitutively expressed Hsp90 accounts for up to 1–2% of the intracellular protein, but may also be present in smaller amounts in circulation. In bullous pemphigoid, anti-BP180 NC16A IgG autoantibodies indirectly lead to enhanced intracellular expression of this protein by generation of an inflammatory response comprising soluble pro-inflammatory mediators and directly cause inhibition of cellular Hsp90 release.

In summary, we demonstrate an upregulated Hsp90 expression at the site of inflammation and an autoantibody-mediated dysregulation of the intracellular and extracellular distribution of Hsp90 in BP patients, as schematically shown in Figure 6. Whether these findings are primary in the pathogenesis of BP or mainly reflect secondary reactive changes induced by inflammation and whether Hsp90 represents a novel and viable target for therapeutic intervention remains to be further explored.
